# Stiffness After Total Knee Arthroplasty: Prevalence and Treatment Outcome

**DOI:** 10.7759/cureus.18271

**Published:** 2021-09-25

**Authors:** Maheswaran Archunan, Girish Swamy, Ashok Ramasamy

**Affiliations:** 1 Trauma and Orthopaedics, Norfolk and Norwich University Hospital, Norwich, GBR; 2 Trauma and Orthopaedics, Colchester General Hospital, Colchester, GBR

**Keywords:** joint stiffness, rehabilitation, pain, manipulation under anaesthesia, post operative complication, arthrofibrosis, total knee arthroplasty

## Abstract

Introduction

Stiffness following total knee arthroplasty (TKA) is an incapacitating complication. The prevalence and causes leading to stiffness are not clearly determined. The aim of the study was to ascertain the prevalence, determine the influencing factors, and evaluate the efficacy of manipulation under anaesthesia (MUA) as a treatment option.

Method

Retrospective review of consecutive series of 1350 primary TKA over a 28-month period. For the purpose of the study, stiffness was defined as flexion contracture of >15 degrees and/or flexion of <75 degrees. Demographic data included co-morbidities, previous knee surgery, pre-operative and post-operative range of movement, anaesthetic techniques and use of nerve blocks, type of prosthesis, ligament balancing including release, mobility post-surgery, patient motivation, physiotherapy, complications, and final range of motion post-MUA.

Results

Of the 1350 patients evaluated, 33 (2.44%) had stiffness defined by the above-outlined criteria and required intervention. Thirty-one patients (2.29%) underwent MUA as a first-line treatment. No complications arose following MUA. One patient (0.07%) required arthroscopic arthrolysis while another patient (0.07%) required revision arthroplasty due to patellar mal-tracking. Following manipulation, mean flexion contracture decreased from 8 degrees to 3.6 degrees, and mean flexion improved from 51.8 degrees to 93.2 degrees. Arc of motion improved in 100% of patients but it is important to note that multiple manipulations were performed in seven patients.

Conclusion

Stiffness after TKA can be difficult to treat and can result in prolonged morbidity and dissatisfaction. This retrospective study highlights the effectiveness of manipulation under anaesthesia as a first-line treatment option leading to improved outcomes especially if done early.

## Introduction

Total knee arthroplasty (TKA) is an established treatment modality for advanced osteoarthritis and has been performed in the UK for the past 50 years. In 2017 alone, more than 100,000 patients underwent TKA procedures in the UK. In approximately 90% of patients, a good or excellent outcome is recorded whereby there is a significant reduction in pain and meaningful improvement in physical function and quality of life [1].

Despite a well-performed surgery, a small percentage of patients, unfortunately, have less favourable outcomes characterised by multiple different issues such as ongoing pain, swelling, instability, and stiffness.

Stiffness also known as arthrofibrosis is infrequent but an incapacitating complication when it occurs. Its prevalence is estimated to be fairly low. In literature, it has been quoted to range between 4% and 16% [2]. There is no universally agreed consensus on the definition of stiffness following TKA. For example, Kim and coworkers defined it as flexion contracture of >15 degrees and/or <75 degrees while Christensen et al. simply defined it as an arc of motion of <70 degrees [3,4].

Furthermore, there are several treatment modalities available to treat patients with stiffness following TKA. This included manipulation under anaesthesia (MUA), arthrolysis (open or arthroscopic), and revision arthroplasty. More recently, there has also been a drive towards the use of intensive physiotherapy as a treatment option but there is little evidence to support this [5]. MUA is both a diagnostic and therapeutic procedure. The surgeon is able to assess the range of movement of the joint and by flexing and extending the joint, it is possible to loosen adhesions to reduce joint stiffness. MUA is thought to be effective when performed within the first three months following the TKA procedure. Beyond this period, surgical procedures such as arthrolysis are likely to give improved outcomes for those with persistent knee stiffness [6,7]. That being said, a systematic review performed by Fitzsimmons et al. has shown that patients can be successfully treated with a combination of arthroscopy and MUA even after one year following their original TKA surgery [8].

In this study, we aimed to determine the prevalence of stiffness following primary knee arthroplasty surgery and to evaluate the results of MUA as the first-line treatment modality.

## Materials and methods

There are no defined criteria for a stiff knee following TKA. However, for the purpose of this study, stiffness was defined as flexion contracture of >10-15 degrees and/or flexion of <75 degrees.

We collected and analysed retrospective data encompassing consecutive series of 1350 primary TKA performed over a 28-month period. Data were collected from theatre records and coding systems.

Demographic data were collected from the three phases of the patients’ treatment journey. Pre-operative data included gender, co-morbidities, previous knee surgery, and pre-operative range of movement. Interoperative data are composed of anaesthetic techniques and nerve blocks, type of prosthesis, and ligament balancing techniques. Post-operative data included a post-op range of movement, mobility post-surgery, patient motivation, physiotherapy, concurrent complications, and final range of motion following MUA.

## Results

Thirty-three patients developed stiffness as defined by the criteria described earlier in this article, equating to a prevalence of 2.44%. Among the patients who developed post-TKA stiffness - 17 patients were male while 16 patients were females. The mean age was 66 years and the mean body weight was 82 kg.

**Figure 1:**
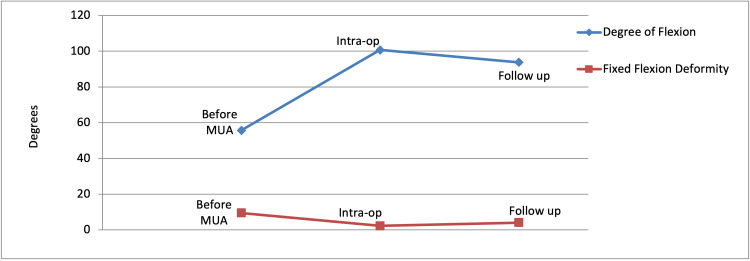
Improvement in the degree of flexion achieved and fixed flexion deformity following MUA MUA: manipulation under anaesthesia

One (0.07%) patient underwent revision knee arthroplasty due to patellar mal-tracking while one other (0.07%) underwent arthroscopic arthrolysis. The remaining 31 (2.29%) patients underwent MUA. There were no complications encountered following the MUA procedures. Following the procedure, the mean degree of flexion improved from 55.7 degrees to 93.8 degrees, and the mean fixed flexion deformity decreased from 9.5 degrees to 4.1 degrees (Table 1 and Figure 1).

**Table 1:** Improvement in the degree of flexion achieved and fixed flexion deformity following MUA MUA: manipulation under anaesthesia

	Before MUA	Intra-op	Follow up
Degree of flexion	55.7°	100.7°	93.8°
Fixed flexion deformity	9.5°	2.3°	4.1°

**Figure 2:**
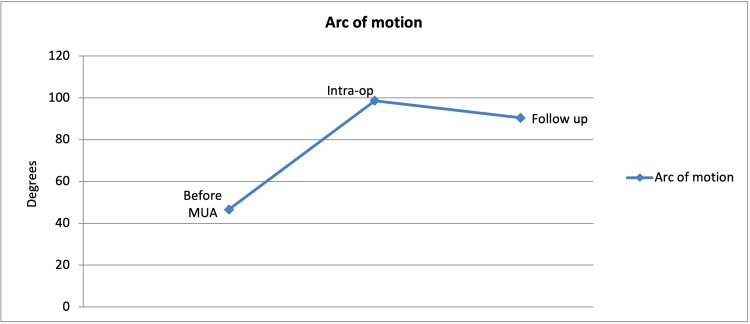
Arc of motion

Furthermore, we are pleased to report that the arc of motion improved in all patients. The mean improvement from pre-MUA to the intra-operative arc of motion was 52.04 degrees (p<0.001), while the mean improvement from pre-MUA to follow-up appointment was 43.81 degrees (p<0.0001; Table 2 and Figure 2).

**Table 2:** Arc of motion

	Before MUA	Intra-op	Follow up
Arc of motion	46.6°	98.6°	90.4°

**Figure 3:**
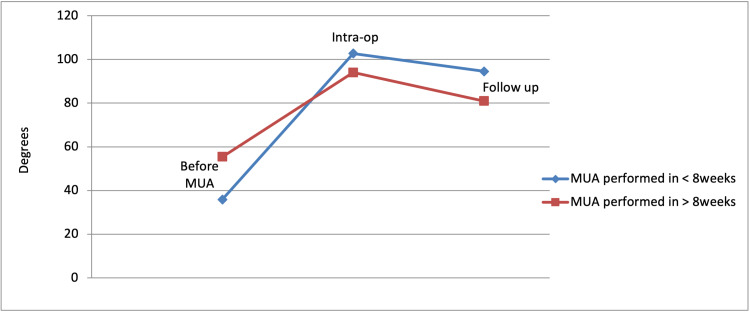
Improved range of motion following MUA: performed >8 weeks vs performed <8 weeks MUA: manipulation under anaesthesia

All patients underwent MUA within 6-15 weeks from their original TKA surgery. Eighteen patients (58%) underwent MUA within eight weeks, while 13 (42%) underwent MUA after eight weeks. Patients who underwent early MUA yielded the best outcomes as their range of movement improved on average by 58.6 degrees while those who underwent MUA after eight weeks saw their range of movement improve by on average by 25.5 degrees (P<0.021; Table 3 and Figure 3).

**Table 3:** Improved range of motion following MUA: performed >8 weeks vs performed <8 weeks MUA: manipulation under anaesthesia

	Before MUA	Intra-op	Follow up
MUA performed in <8 weeks	35.9°	102.7°	94.5°
MUA performed in >8 weeks	55.4°	94°	80.9°

**Figure 4:**
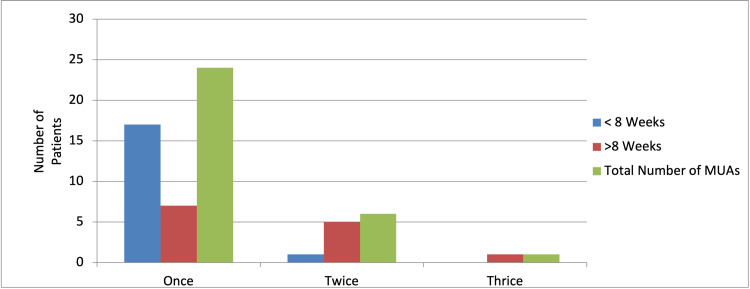
Number of MUA required by patients MUA: manipulation under anaesthesia

However, seven patients underwent multiple MUA before achieving satisfactory results. Six patients required MUA twice while one patient required it three times. Although it is important to note that 86% of patients who required more than one MUA had their index procedure performed more than eight weeks earlier (Table 4 and Figure 4).

**Table 4:** Number of MUA required by patients MUA: manipulation under anaesthesia

	Once	Twice	Thrice
<8 weeks	17	1	0
>8 weeks	7	5	1
Total number of MUAs	24	6	1

## Discussion

Development of stiffness following TKA has been attributed to have a multifactorial nature rather than a single issue. These can be subdivided into preoperative, intraoperative, and post-operative causes. Preoperative factors include patient co-morbidities, lifestyle habits, and significantly poor baseline range of movement of the affected joint prior to their knee replacement surgery. Intra-operative causes may include malrotation of components, incorrect sizing of components, and failure to balance sagittal gaps. Postoperative factors include poor compliance, pain, extensive fibrosis, and heterotrophic bone formations which may mechanically block movement. Furthermore, the infection should be considered in those who progressively deteriorate in their range of motion [9]. It must be recognised that MUA is likely to only be effective in patients who have developed stiffness because of intraarticular adhesions [10].

Within the 28-month period we evaluated, 1350 suitable patients were identified. All patients underwent primary TKA for osteoarthritis. The prevalence of stiffness following TKA was found to be 2.44%. This figure is proportional when compared to other series such as by Kim and coworkers revealing a prevalence of 1.3% while Yercan et al. found a prevalence of 5.3% [3,11].

Our study shows that significant results can be achieved with MUA. Improvement in patient outcomes was seen in all three aspects - increase in the arc of motion, increase in the degree of flexion, and reduction of fixed flexion deformity. This of course directly correlates with improved patient satisfaction. Furthermore, MUA is a relatively straightforward procedure with few associated risks. Possible risks include periprosthetic fracture, wound breakdown, bleeding, failed procedure, and risks associated with general anaesthesia. We are pleased to report that none of the patients who underwent MUA in our study developed any early or late complication post-procedure.

The findings of our study certainly confirm that performing the MUA procedure earlier yielded more favourable results. The patients had a greater improvement in their overall knee range of motion and were also less likely to require repeat MUA procedures to help alleviate their symptoms. In our study, 58% of patients underwent the procedure within eight weeks of their initial knee replacement surgery. In view of these findings, it is important to highlight that MUA should be offered to patients promptly in their post-operative period if they were to be suffering from substantial joint stiffness.

A similar case series was performed by Mohammed et al. in 2009 consisting of 519 patients who underwent either total or unicompartmental knee replacement. The prevalence of stiffness was found to be 4%. Following MUA, the mean arc of motion improved from 60.2 degrees to 91.9 degrees. The author concluded that MUA was a satisfactory treatment modality [10]. Although this study consisted of fewer patients when compared to ours, the outcomes are very much comparable as our mean arc of motion improved from 46.6 degrees to 90.4 degrees. Furthermore, case series performed by Yercan et al. in 2006 consisting of 1188 patients concluded that those who underwent early MUA benefitted with superior outcomes when compared to those who had the MUA procedure later in the postoperative period [11]. This finding is very much supported by the data obtained in our study.

The limitations of this study include the accuracy of the retrospective data that were collected and inter-observation bias. The entirety of the data is retrospective; therefore, the quality and accuracy of the data collection are dependent on how well medical documentation were originally recorded. Furthermore, the outcomes of the MUA procedure can be subjected to inter-observer bias when the data were originally recorded by the surgeon. Finally, the outcomes of this study could be further strengthened by the patient-reported outcome measures tool.

## Conclusions

This study reaffirms that very good results can be achieved with MUA when treating patients who have developed stiffness following their knee arthroplasty procedure. It resulted in improved fixed flexion deformity and improved range of motion. Furthermore, there were no significant complications encountered following the MUA procedures. We, therefore, put forward, that when treating patients with stiffness following TKA, MUA is an excellent first-line treatment option, especially when performed early.
